# Non-linear kernel methods for genomic prediction of soybean yield and quality in multi-environment trials

**DOI:** 10.1007/s00122-026-05387-3

**Published:** 2026-09-23

**Authors:** Wanessa Alves Lima Paiva, Weverton Gomes da Costa, Leandro Pacheco Machado, Ana Carolina Campana Nascimento, Diego Jarquin, Moyses Nascimento

**Affiliations:** 1 Laboratory of Computational Intelligence and Statistical Learning (LICAE), Department of Statistics, Federal University of Viçosa, Viçosa, Minas Gerais Brazil; 2 Agronomy Department, University of Florida, Gainesville, FL USA

## Abstract

**Key message:**

Nonlinear kernels improve the accuracy of genomic prediction while preserving the inferential framework of quantitative genetics.

**Abstract:**

Improving the predictive accuracy of genomic prediction (GP) for complex genetic architectures involving non-additive effects and genotype-by-environment interactions (GEI) requires alternative approaches beyond conventional linear models such as Genomic Best Linear Unbiased Prediction (GBLUP). In this study, we evaluated grain yield, protein content, and oil content in the SoyNAM population, comprising 1,379 genotypes evaluated across six environments, to compare four non-linear kernels (Laplacian, Gaussian, Bessel, and Polynomial) with the GBLUP and Random Forest a Machine Learning model. Two variance structures (main effects and main plus interaction effects) were evaluated under three cross-validation schemes: CV1 (predicting untested genotypes in observed environments), CV2 (predicting tested genotypes in observed environments), and CV0 (predicting tested genotypes in unobserved environments). In addition, we tested the same models in a epistatic trait simulated dataset. The inclusion of GEI effects substantially improved predictive accuracy for grain yield under CV1 and CV2, whereas only modest gains were observed for protein and oil content. Across traits and validation schemes, the nonlinear kernels consistently matched or outperformed GBLUP, with the greatest advantage observed for oil content and under the CV0 scheme. No single nonlinear kernel consistently outperformed the others, indicating that kernel performance was environment dependent. Compared with Random Forest, the non-linear kernels achieved comparable or superior predictive accuracy while preserving the mixed-model framework, enabling the estimation of variance components. Moreover, in the extra dataset, the Laplacian kernel consistently showed high predictive accuracy across all scenarios, supporting its use as a promising option to the widely used Gaussian kernel. Therefore, non-linear kernel methods provide a robust and interpretable alternative for GP.

**Supplementary Information:**

The online version contains supplementary material available at https://doi.org/10.1007/s00122-026-05387-3.

## Introduction

Many important agronomic traits are governed by multiple quantitative trait loci (QTLs) and exhibit complex genetic architectures influenced by distinct genetic effects and several environmental factors (Mackay et al. 2009; Kumar et al. 2017). Such complexity poses challenges for understanding the genetic basis of these traits and accurately predicting phenotypic performance (Cooper et al. 2009; van Eeuwijk et al. 2010).

The soybean [*Glycine max* (L.) Merr.] is an economically important self-pollinated crop worldwide, valued for its high protein and oil content and its central role in food, feed, and biofuel production (Hartman et al. 2011). In this crop, complex traits such as grain yield, 100-seed weight, fatty acid composition, seed protein and oil contents, are determined by additive and additive × additive epistatic effects (AAE), with multiple QTLs contributing to trait expression across different environmental conditions (Barona et al. 2012; Fan et al. 2015; Duhnen et al. 2017; Li et al. 2020). When several QTLs interact strongly with the environment, trait expression becomes highly dependent on environmental conditions, a phenomenon known as Genotype-by-Environment Interaction (GEI) (Wang et al. 2020).

In this context, Nested Association Mapping (NAM) populations are particularly effective for studying self-pollinated crops that rely on inbred lines, where the observed genetic variation arises primarily from additive and AAE effects. This occurs because successive generations of selfing lead to near-complete homozygosity, effectively eliminating dominance effects (Cook et al. 2012; Ellis et al. 2023). By capturing genetic variation across diverse genetic backgrounds, NAM populations provide a robust framework for training Genomic Prediction (GP) models (Gireesh et al. 2021). Furthermore, when these lines are evaluated in Multi-Environment Trials (METs), the capacity of models to account for GEI and predict genotypic performance in untested environments significantly enhances the stability and transferability of predictions (Persa et al. 2022).

GP models aim to optimize selection accuracy by accounting for both linear (additive) and non-linear or interactive effects (AAE and GEI) (Mackay 2014; Fritsche-Neto et al. 2025). However, capturing these non-linear genetic interactions effectively requires advanced modeling strategies. Howard et al. (2014) demonstrated that, when only additive effects are present, parametric (Least Squares Regression, Ridge Regression, Bayesian Ridge Regression, Least Absolute Shrinkage and Selection Operator (LASSO), Bayesian LASSO, Best Linear Unbiased Prediction (BLUP), Bayes A, Bayes B, Bayes C, and Bayes Cπ) and nonparametric (Nadaraya-Watson estimator, Reproducing Kernel Hilbert Space (RKHS), Support Vector Machine, and Neural Networks) statistical methods exhibit similar performance in terms of prediction accuracy in GP. However, when epistasis is present, nonparametric methods generally outperform. This pattern was also related in the studies of Barbosa et al. (2021) and Costa et al. (2022). Thereby, the use of more advanced modeling strategies, such as non-linear kernel methods and Machine Learning techniques, is required to effectively capture complex, non-linear genetic interactions and improve predictive performance.

Ray et al. (2023) compared the conventional parametric model, the Genomic Best Linear Unbiased Predictor (GBLUP) (a linear kernel weighted for allele frequency), against three nonparametric methods (Deep Learning, Arc-Cosine kernel, and Gaussian kernel) to predict traits of soybean in MTEs. GBLUP presented better or similar results than those obtained by the nonparametric methods. According to these authors, the result can be in part explained by the possibility of suboptimal hyperparameters in the nonparametric models. However, it is important to acknowledge the existence of a diversity of additional non-linear kernels in the literature (beyond Gaussian and Arc-Cosine kernels) which diverge in their mathematical formulation and, crucially, in their capacity to model non-linear AAE and GEI.

Therefore, the evaluation of these alternative non-linear kernels is essential, as they represent the potential to surpass the performance of the conventional GBLUP model. This study aims to evaluate whether diverse kernel methods, jointly with hyperparameter optimization, can outperform the GBLUP model in the genomic prediction of Grain Yield, Protein Content, and Oil Content within the SoyNAM subpopulation across six environments.

## Materials and methods

### Phenotypic data

The Soybean NAM (SoyNAM) population is publicly available through the SoyBase (https://soybase.org/) (Song et al. 2017; Xavier et al. 2018) and it comprises approximately 5,400 RILs derived from 40 biparental families, each generated by crossing a genetically diverse founder line with a common elite parent (Song et al. 2017; Xavier et al. 2018, 2022). All lines were genotyped using the SoyNAM 6 K BreadChip SNP array, providing dense genome-wide coverage for QTL discovery and genomic prediction. In this study, we used the already processed SoyNAM dataset conveniently selected in Ray et al. (2023). This data set includes a subset of 1,379 genotypes evaluated across six environments for grain Yield, Protein, and Oil content. Environments were defined as location × year combinations from trials conducted in Iowa - IA (2012), Illinois - IL (2011 and 2012), Indiana - IN (2012), and Nebraska - NE (2011 and 2012). All genotypes were tested in each environment, resulting in a total of 8,274 phenotypic records per trait (1,379 genotypes × 6 environments).

In this dataset, the micro-environmental and design variability for each trait within each environment was previously controlled using the correction framework of the SoyNAM package, generating the adjusted phenotypic records (**y**) utilized as inputs for the multi-environment genomic models. Moreover, after filtering out markers with a minor allele frequency (MAF) below 0.05, a total of 4,325 Single Nucleotide Polymorphisms (SNPs) were retained for analysis.

### Cross-validation schemes

To evaluate model performance, different cross-validation (CV) schemes were applied, following the approach described by Ray et al. (2023). In this framework, CV1 represents a breeding scenario in which unobserved individuals are predicted, CV0 corresponds to the prediction of unobserved environments, and CV2 reflects an unbalanced scenario in which individuals are not evaluated across all locations. For each CV scheme, prediction accuracy was quantified using the Pearson correlation coefficient between observed and predicted values.

### Statistical methods

To predict grain Yield, Protein, and Oil content of soybeans, we used different relationship matrices in the mixed model equations, according to the structures:

#### Main effects

$$\:\boldsymbol{y}\:=\:\mu\:1\:+\:{\boldsymbol{Z}}_{e}\boldsymbol{E}\:+\:{\boldsymbol{Z}}_{g}\:{\boldsymbol{u}}_{g}\:+\:\boldsymbol{\epsilon\:}$$

where **y** is the vector of phenotypic records of *l* genotypes evaluated in *a* environments (*l* x a = n), $\:\mu\:$ is a common effect, **1** is a vector composed of 1’s in all elements, **E** is the vector of fixed environmental effects, and $\:{\boldsymbol{Z}}_{e}$ is the incidence matrix that links environments to the phenotypic observations. The genetic vector $\:{\boldsymbol{u}}_{g}$ **~ N(0**,** G**$\:{\sigma\:}_{g}^{2}$**)**, where **G** is the genomic relationship matrix constructed using different approaches, including GBLUP and kernel-based methods, $\:{\boldsymbol{Z}}_{g}$ is the incidence matrix that connects genotypes with phenotypic records, and $\:{\sigma\:}_{g}^{2}$ the genetic variance component. The error term was assumed as **ε ~ N(0**,** I**_n_$\:{\sigma\:}_{\epsilon\:}^{2}$**)**, capturing error variability not explained by the other terms.

#### Main and interaction effects

$$\:\boldsymbol{y}\:=\:\mu\:1\:+\:{\boldsymbol{Z}}_{e}\boldsymbol{E}\:+\:{\boldsymbol{Z}}_{g}\:{\boldsymbol{u}}_{g}\:+\:{\boldsymbol{Z}}_{ge}{\boldsymbol{u}}_{ge}\:+\:\boldsymbol{\epsilon\:}$$

where $\:{\boldsymbol{u}}_{ge}$ is the multiplicative effect that represents the interaction between genotypes by environment combinations, with $\:{\boldsymbol{u}}_{ge}$ **~**$\:\boldsymbol{N}\left(0,\left[{\boldsymbol{Z}}_{g}\boldsymbol{G}{\boldsymbol{Z}}_{g}^{T}\right]\odot\:\left[{\boldsymbol{Z}}_{e}{\boldsymbol{I}}_{a}{\boldsymbol{Z}}_{e}^{T}\right]{\sigma\:}_{ge}^{2}\right)$ and $\:{\boldsymbol{Z}}_{ge}$ equal to $\:{\boldsymbol{Z}}_{g}\bigotimes{\boldsymbol{Z}}_{e}$ representing the incidence matrix that links the interaction between the genotypes and environments to the phenotypic observations. The remaining terms were assumed as previously described.

Although the environment-specific interaction component does not induce direct covariance between environments when an identity matrix is used for the environmental dimension, its inclusion is important because it explicitly models genotype-specific deviations from the overall genomic effect within each environment. Thus, the common genomic main effect captures information shared across environments, whereas the interaction component accounts for environment-specific departures from this common response. This formulation is conceptually consistent with the marker × environment GBLUP framework described by López-Cruz et al. (2015), in which the genomic relationship structure is combined with an environmental identity matrix to model environment-specific genomic effects.

For both models, the GBLUP and non-linear kernel methods were implemented.

#### GBLUP method

In the GBLUP method, the **G** matrix was calculated using the codification proposed by VanRaden (2008), using the AGHmatrix (Amadeu et al. 2023) R package (R Core Team 2025):

$$\:\boldsymbol{G}\:=\:\frac{\boldsymbol{W}{\boldsymbol{W}}^{T}}{2{\varSigma}_{j}{p}_{j}(1\:-{p}_{j})}$$

where **W** is the centered markers matrix, and *p*_*j*_ is the allele frequency at the *j*^th^ marker.

#### Non-linear kernel methods

In non-linear kernel methods (Laplacian, Gaussian, Bessel, and Polynomial), the $\:\boldsymbol{G}$ matrix was obtained by applying a kernel function $\:k(\cdot\:,\cdot\:)$ to the marker matrix, such that each element $\:{\boldsymbol{G}}_{ij}=k\left({\boldsymbol{x}}_{i},\:{\boldsymbol{x}}_{j}\right)$ represents the genomic similarity between genotypes *i* and *j*, computed as a function of their marker profiles $\:{\boldsymbol{x}}_{i}$ and $\:{\boldsymbol{x}}_{j}$, using the kernlab (Karatzoglou et al. 2004) R package (R Core Team 2025).

The performance of kernel functions can vary substantially depending on the chosen hyperparameters. Hyperparameters are configuration variables defined before model training that govern both the learning process and the structural characteristics of the algorithms (Ali et al. 2023). Therefore, for each kernel, an empirical grid search strategy was used to evaluate different levels of kernel smoothness and complexity. The possible combinations within the predefined ranges were evaluated independently for each model.

For the Gaussian and Laplacian kernels, the bandwidth parameter sigma ($\:\sigma\:$**)** controls the decay of genetic similarity with increasing genotype distance (Hu et al. 2023). Thus, the evaluated values $\:\sigma\:$ *ε* {0.0001, 0.001, 0.01, 0.1} represented different levels of kernel smoothness. To assess whether these bandwidth values produced degenerate kernel matrices, the distribution of the off-diagonal elements of the Gaussian and Laplacian kernel matrices was inspected (Pérez and de los Campos 2014). The Laplacian and Gaussian kernels were respectively defined as:

$$\:k({\boldsymbol{x}}_{i},\:{\boldsymbol{x}}_{j})\:=\:{e}^{-\sigma\:\left|\right|{\boldsymbol{x}}_{i}\:-\:{\boldsymbol{x}}_{j}\left|\right|}$$

$$\:k({\boldsymbol{x}}_{i},\:{\boldsymbol{x}}_{j})\:=\:{e}^{-\sigma\:\left|\right|{\boldsymbol{x}}_{i}\:-\:{\boldsymbol{x}}_{j}|{|}^{2}}$$

For the Bessel kernel, the evaluated grid was designed to explore a restricted and computationally feasible range of non-linear distance-based similarity structures. In this kernel, $\:\sigma\:$ controls the scale of the distance effect, whereas the degree and order parameters modify the shape and flexibility of the kernel function. Low-degree and low-order values were evaluated to allow non-linear patterns while reducing the risk of overly complex kernels and unstable model comparison. Therefore, we tested degree (d) *ε* {2, 3}; sigma (σ) *ε* {0.001, 0.01, 0.1}; order (*J*) *ε* {0, 1} according to:

$$\:k\left({\boldsymbol{x}}_{i},\:{\boldsymbol{x}}_{j}\right)\:=\:\frac{J\:\left(\sigma\:\left|\left|{\boldsymbol{x}}_{i}\:-\:{\boldsymbol{x}}_{j}\right|\right|\right)}{{\left(\sigma\:\left|\left|{\boldsymbol{x}}_{i}\:-\:{\boldsymbol{x}}_{j}\right|\right|\right)}^{d}}$$

For the polynomial kernel, the evaluated grid was defined to assess low-order non-linear marker relationships. The degree parameter controls the order of the non-linear relationship, while the scale and offset parameters control the magnitude of the inner-product term and the contribution of lower-order components (Schölkopf and Smola 2002; Hastie et al. 2009). Degrees 2 and 3 were used to represent quadratic and cubic similarity structures, while avoiding high-degree polynomial kernels that could inflate kernel values and increase the risk of overfitting. Thereby, we tested degree (*d*) *ε* {2, 3}; scale (*sc*) *ε* {0.1, 1}; offset (*off*) *ε* {0, 1} on:

$$\:k({\boldsymbol{x}}_{i},\:{\boldsymbol{x}}_{j})\:=\:({sc\:\langle{\boldsymbol{x}}_{i},\:{\boldsymbol{x}}_{j}\rangle\:+off)}^{d}$$

**Fig. 1:**
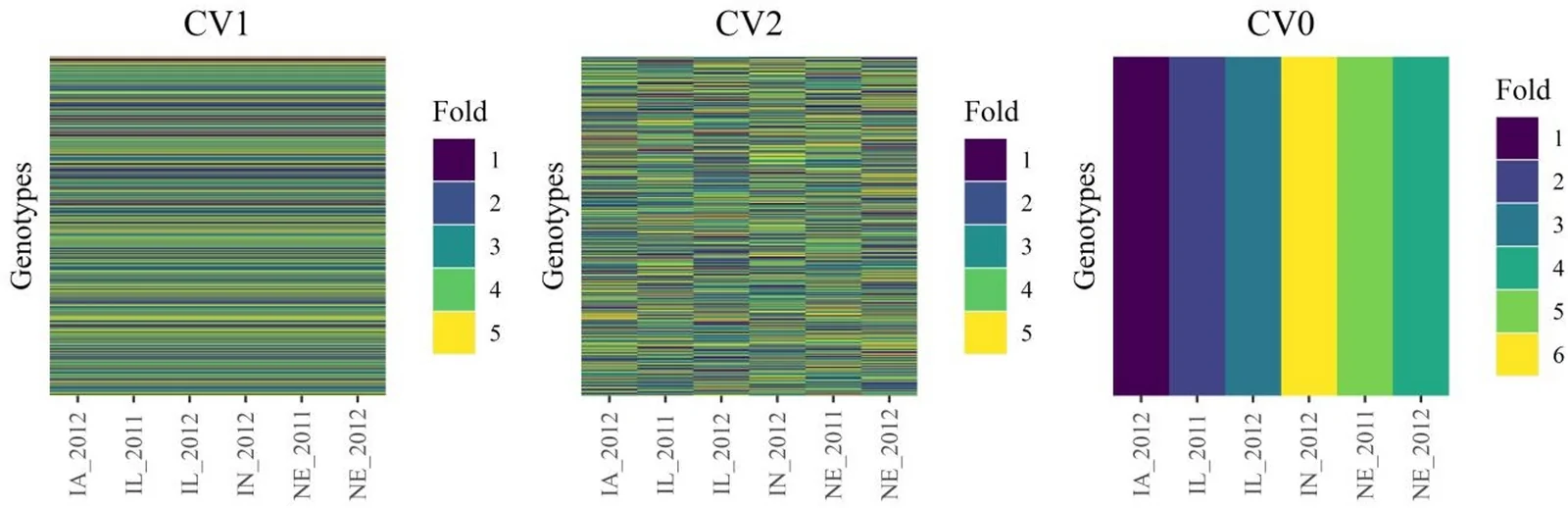
Schematic representation of three cross-validation schemes (CV1, CV2, and CV0) for phenotypic prediction

For each kernel, the optimal hyperparameter combination was selected based on prediction accuracy in the main effects framework performance across cross-validation schemes (Fig. 1). This approach allows the effect of each hyperparameter to be evaluated without the additional complexity of the GEI, which may capture part of the variation and mask differences among hyperparameter settings. Moreover, use the average across cross-validation schemes provides a more robust measure of overall hyperparameter performance and reduces dependence on a single validation scheme.

Representative shapes of the kernels tested, illustrating the influence of different hyperparameter combinations on kernel behavior, are provided in the Supplementary Figs. 1–3. Finally, the kernels with the best hyperparameter combinations were compared with GBLUP under the main effects and main plus interaction frameworks, with final predictive accuracies for CV1 and CV2 reported as the mean across five random splits.

### Variance components

Using all available data, genetic ($\:{\sigma\:}_{g}^{2}$), genotype-by-environment interaction ($\:{\sigma\:}_{ge}^{2}$) and error ($\:{\sigma\:}_{\epsilon\:}^{2}$) variance components were estimated across the different models for Yield, Protein, and Oil. Based on these variance components, broad-sense heritability (proportion of phenotypic variance explained by genetic component) was calculated as $\:{H}^{2}$ = $\:\frac{{\sigma\:}_{g}^{2}}{{\sigma\:}_{g}^{2}\:+\:{\sigma\:}_{\epsilon\:}^{2}}$ for models considering only main effects, and as $\:{H}^{2}$ = $\:\frac{{\sigma\:}_{g}^{2}}{{\sigma\:}_{g}^{2}\:+\:{\sigma\:}_{ge}^{2}\:+\:{\sigma\:}_{\epsilon\:}^{2}}$ for models including both main and interaction effects.

### Data analysis

All models were fitted using the RKHS method implemented in the BGLR (Pérez and De Los Campos 2014) R package (R Core Team 2025). Each model was run for 20,000 iterations, with a burn-in of 5,000 and a thinning interval of 5.

### Machine learning comparison

To provide a comparison with a representative Machine Learning approach, Random Forest was implemented using the ranger package in R (R Core Team 2025). SNP markers were used directly as predictor variables, while the environment was included as a categorical. The Random Forest model consisted of 500 decision trees, with the number of variables randomly sampled at each split (mtry) set to the square root of the total number of predictor variables and the minimum terminal node size set to five observations. To ensure a fair comparison with the kernel-based models, the same cross-validation schemes were adopted, and for CV1 and CV2 the reported predictive accuracies correspond to the mean across five independent random data partitions.

### Validation on an independent dataset

To evaluate the predictive accuracy of non-linear kernels in a dataset known to contain epistatic effects, we used the epistatic dataset previously analyzed by Costa et al. (2026). It consisted of a simulated F₂ population diploid specie (2n = 2x = 20) with 1,000 individuals and 4,010 codominant SNP markers distributed across 10 linkage groups. Six traits were simulated under epistatic architectures, differing in the number of QTLs controlling the trait: 8, 40, 80, 120, 240, and 480. Heritability was set to 0.70 for the traits with 8 and 40 QTLs, 0.50 for those with 80 and 120 QTLs, and 0.30 for those with 240 and 480 QTLs.

We evaluated the same models used for the soybean dataset. First, hyperparameter tuning was performed separately for each non-linear kernel. Then, the Laplacian, Gaussian, Bessel, and Polynomial kernels with their best hyperparameter configurations were compared with GBLUP and Random Forest. As the epistatic dataset consisted of single-environment traits, predictive accuracy was assessed using five-fold cross-validation. To evaluate the stability of model performance, the cross-validation procedure was repeated across five independent data splits.

A repository containing all the scripts and documentation to reproduce the analysis is available at: https://wlp.quarto.pub/soybean-traits-prediction/.

## Results

### Phenotypic analysis

Mean grain yield across environments ranged from 2,781 (IA_2012) to 5,174 kg·ha⁻¹ (NE_2011), with standard deviations between 469 and 889 kg·ha⁻¹ (Supplementary Fig. 4A). Phenotypic correlations for Yield were generally low to moderate, peaking between NE_2011 and NE_2012 *r* ≈ 0.46), while IL_2012 remained consistently decoupled from other sites *r* ≤ 0.15 (Supplementary Fig. 4B).

For seed composition, protein content averaged 32.6–34.8% and oil content 18.8–20.4% across all environments (Supplementary Figs. 5 A, 6 A). Both traits showed stronger environmental stability than yield, with high correlations observed between NE_2011 and NE_2012 (*r* = 0.70 for Protein; *r* = 0.75 for Oil). In both cases, IN_2012 exhibited the weakest correlations with remaining environments (Supplementary Figs. 5B, 6B).

### Hyperparameter determination kernels

The predictive performance of the Laplacian, Bessel, Polynomial, and Gaussian kernels for grain yield, protein, and oil content in soybean varied according to the hyperparameters used, as well as across cross-validation schemes and environments (Supplementary Figs. 1–3).

**Fig. 2:**
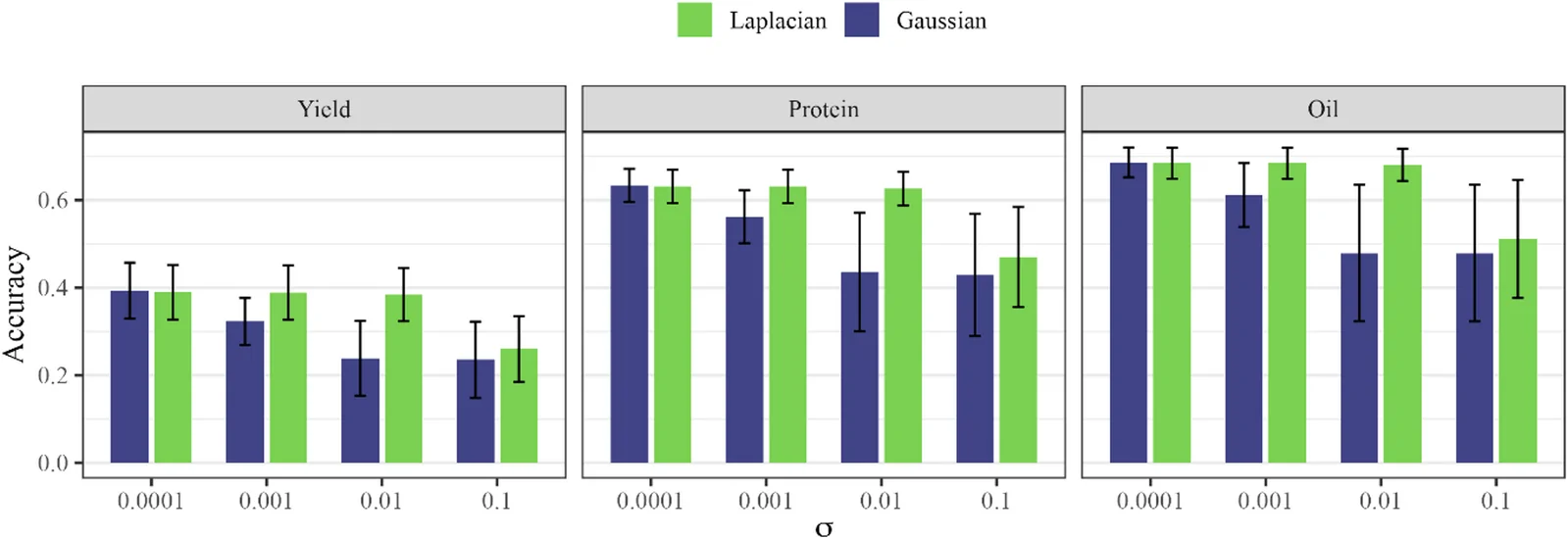
Mean predictive accuracy across cross-validation schemes and environments considering only main effects for genomic prediction models using Laplacian and Gaussian kernels under different bandwidth (σ) values for the traits Yield, Protein, and Oil

The predictive accuracy of the Laplacian and Gaussian kernels varied according to the bandwidth parameter (σ) across grain Yield, Protein, and Oil content (Fig. 2). For the Gaussian kernel, mean predictive accuracy generally decreased as σ increased, with the highest accuracies consistently obtained at σ = 0.0001 for all three traits. The Laplacian kernel was less sensitive to changes in σ, maintaining similar mean predictive performance between σ = 0.0001 and 0.01 for all three traits, whereas a reduction was observed at σ = 0.1. Considering the minimal differences in predictive performance among the best-performing bandwidths and favoring the least complex parameterization, σ = 0.0001 for the Gaussian kernel and σ = 0.001 for the Laplacian kernel were selected for all subsequent analyses.

**Fig. 3:**
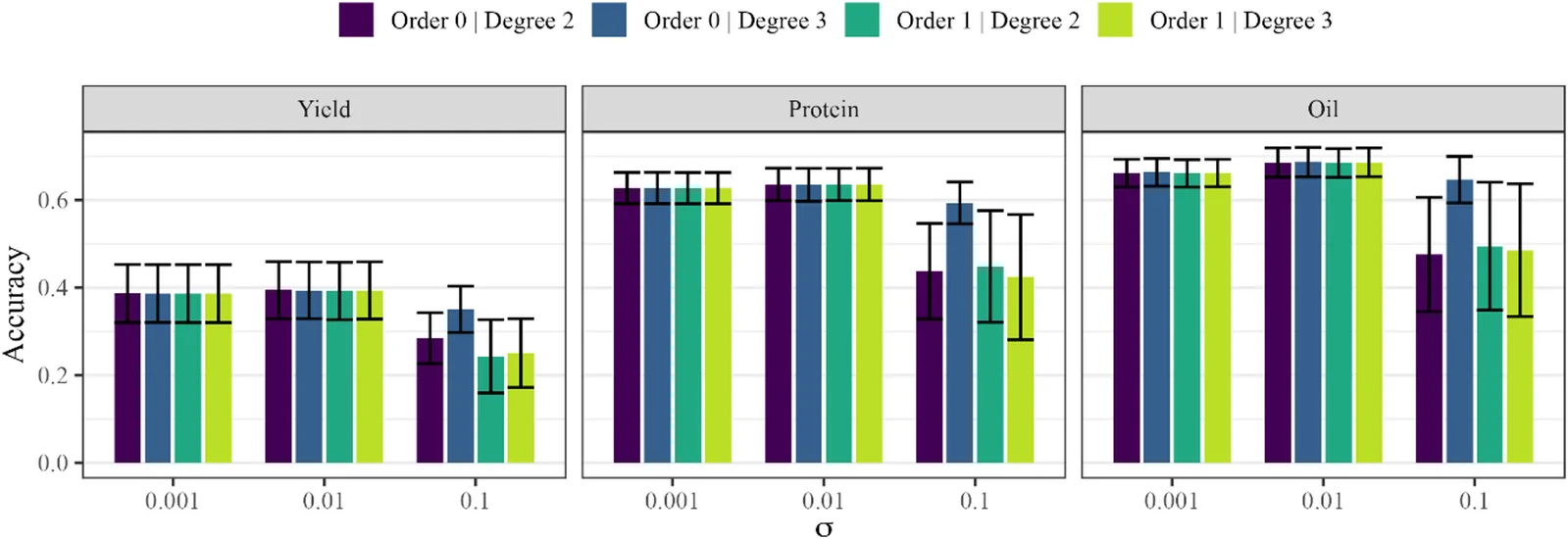
Mean predictive accuracy across cross-validation schemes and environments considering only main effects for genomic prediction models using Bessel kernel under different sigma, degree, and order values for the traits Yield, Protein, and Oil

The Bessel kernel performance was strongly influenced by the σ parameter across all evaluated traits (Yield, Protein, and Oil) (Fig. 3). Lower σ values consistently produced higher and more stable mean accuracies, whereas higher σ (0.1) led to a marked reduction in performance for all parameter combinations. The effect of the degree and order parameters was relatively small, showing very similar behavior and no consistent superiority between them. Thus, degree = 2, σ = 0.01, and order = 0 were selected for all subsequent analyses to favor a more parsimonious model.

**Fig. 4:**
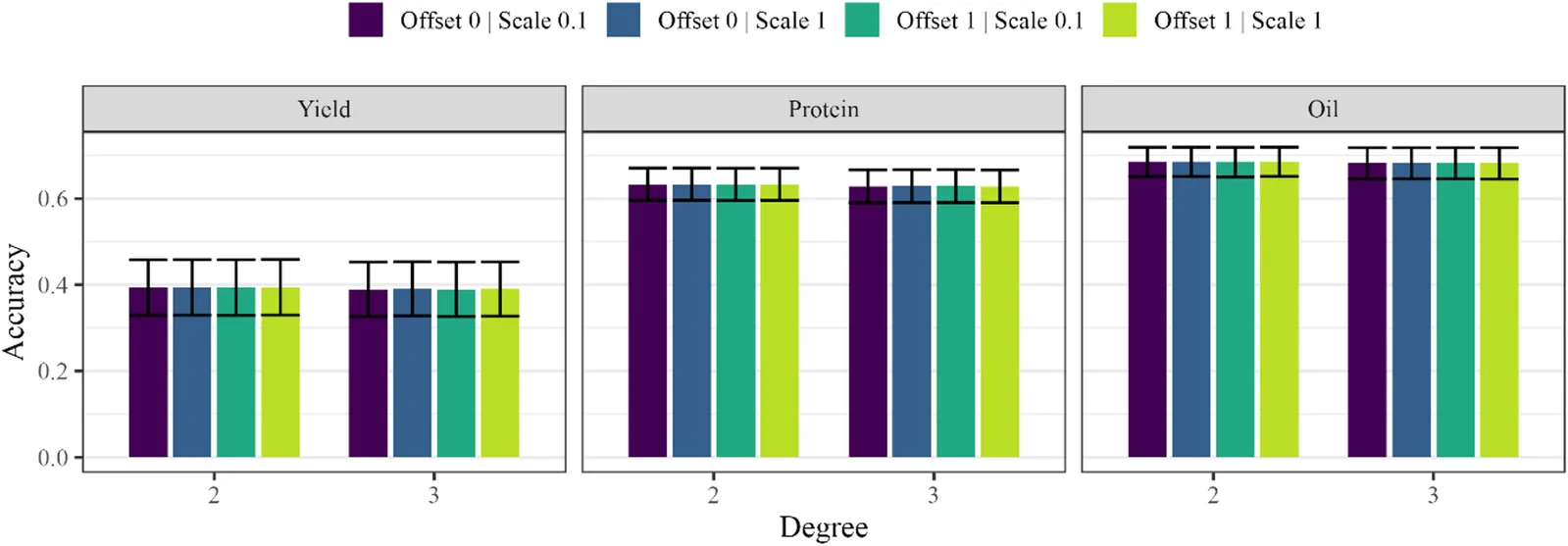
Mean predictive accuracy across cross-validation schemes and environments considering only main effects for genomic prediction models using Polynomial kernel under different degree, offset, and scale values for the traits Yield, Protein, and Oil

Finally, across all traits (Yield, Protein, and Oil), the Polynomial kernel showed little sensitivity to the combinations of degree, scale and offset, with nearly identical mean predictive accuracies across all evaluated settings. Therefore, to favor a more parsimonious parameterization, degree = 2, scale = 1, and offset = 1 were selected for all subsequent analyses Fig. 4.

### Variance components

**Fig. 5:**
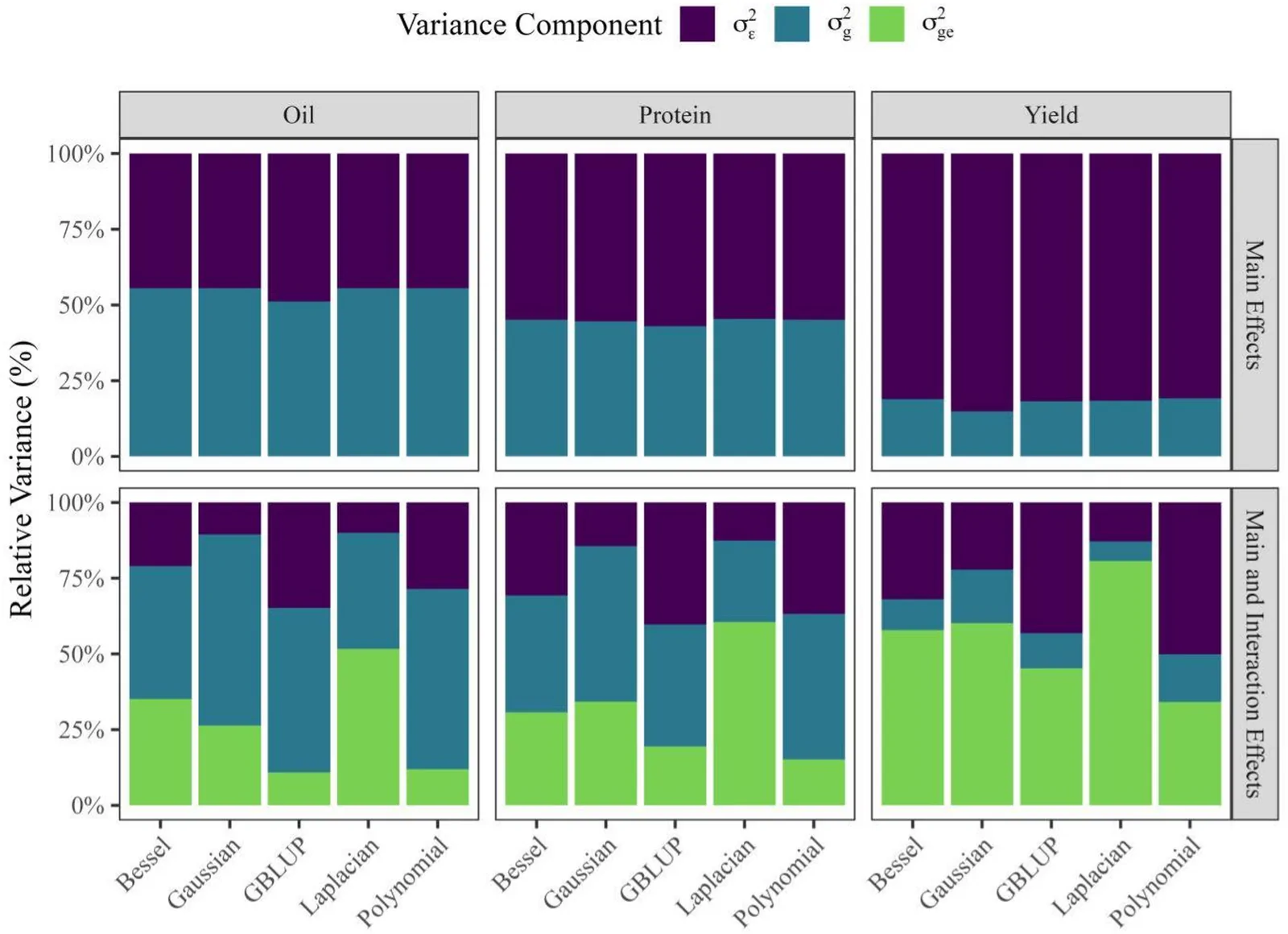
Percentage of variability explained by variance components estimates for Yield, Protein, and Oil across different models (GBLUP, Laplacian, Gaussian, Bessel, and Polynomial), considering the effects included in each model, without cross-validation or environment-specific stratification

Variance components partitioning varied across traits and models (Supplementary Table 1; Fig. 5). Across all evaluated kernel models, the genetic component ($\:{\sigma\:}_{g}^{2}$) accounted for a smaller proportion of the total variance in Yield (17.9% on average) compared to Protein (44.7%) and Oil (54.7%) contents. Consequently, the error variance ($\:{\sigma\:}_{\epsilon\:}^{2}$) was the predominant factor for Yield (82.1%) under this simplified model. When the GEI were incorporated, the variance distribution shifted. For Yield, $\:{\sigma\:}_{\boldsymbol{g}\boldsymbol{e}}^{2}$ became the major contributor (55.6%), while and $\:{\sigma\:}_{\epsilon\:}^{2}$ accounted for 12.3% and 32.0%, respectively. In contrast, for seed composition traits, $\:{\sigma\:}_{\boldsymbol{g}}^{2}$ remained robust; for Protein, (41.0%) was comparable to $\:{\sigma\:}_{\boldsymbol{g}\boldsymbol{e}}^{2}$, whereas for Oil, $\:{\sigma\:}_{\boldsymbol{g}}^{2}$ (51.8%) remained the primary variance component, even with the inclusion of $\:{\sigma\:}_{\boldsymbol{g}\boldsymbol{e}}^{2}$ (27.2%) and $\:{\sigma\:}_{\epsilon\:}^{2}$ (21.0%).

**Fig. 6:**
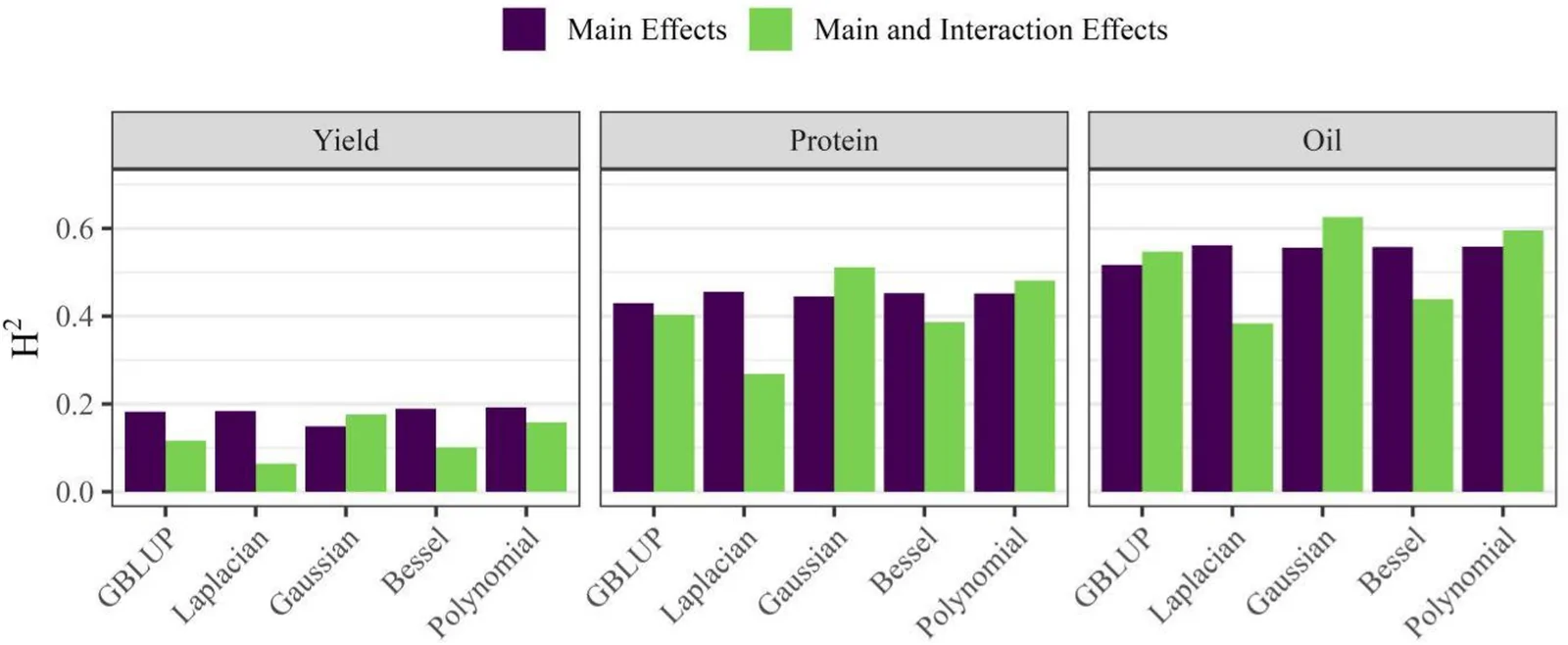
Broad sense heritability ($\:{H}^{2}$) estimates for Yield, Protein, and Oil across different models (GBLUP, Laplacian, Gaussian, Bessel, and Polynomial), considering the effects included in each model, without cross-validation or environment-specific stratification

Estimates of *H*^*2*^ varied across traits and models (Fig. 6). For Yield, $\:{H}^{2}$ averaged 0.179 ± 0.0162 when the main effects were considered, decreasing to 0.123 ± 0.0414 with the inclusion of the GEI effect. Seed composition traits exhibited higher and more stable heritability. For Protein, the mean $\:{H}^{2}$ was 0.447 ± 0.009 under main effects, showing a slight decrease to 0.410 ± 0.087 when interaction effects were incorporated into the model. Similarly, for Oil, the mean $\:{H}^{2}$ was 0.550 ± 0.017 when considering only main effects, decreasing to 0.518 ± 0.095 with the inclusion of main and interaction effects.

### Predictive accuracy across cross-validation scenarios

#### Grain yield

**Fig. 7:**
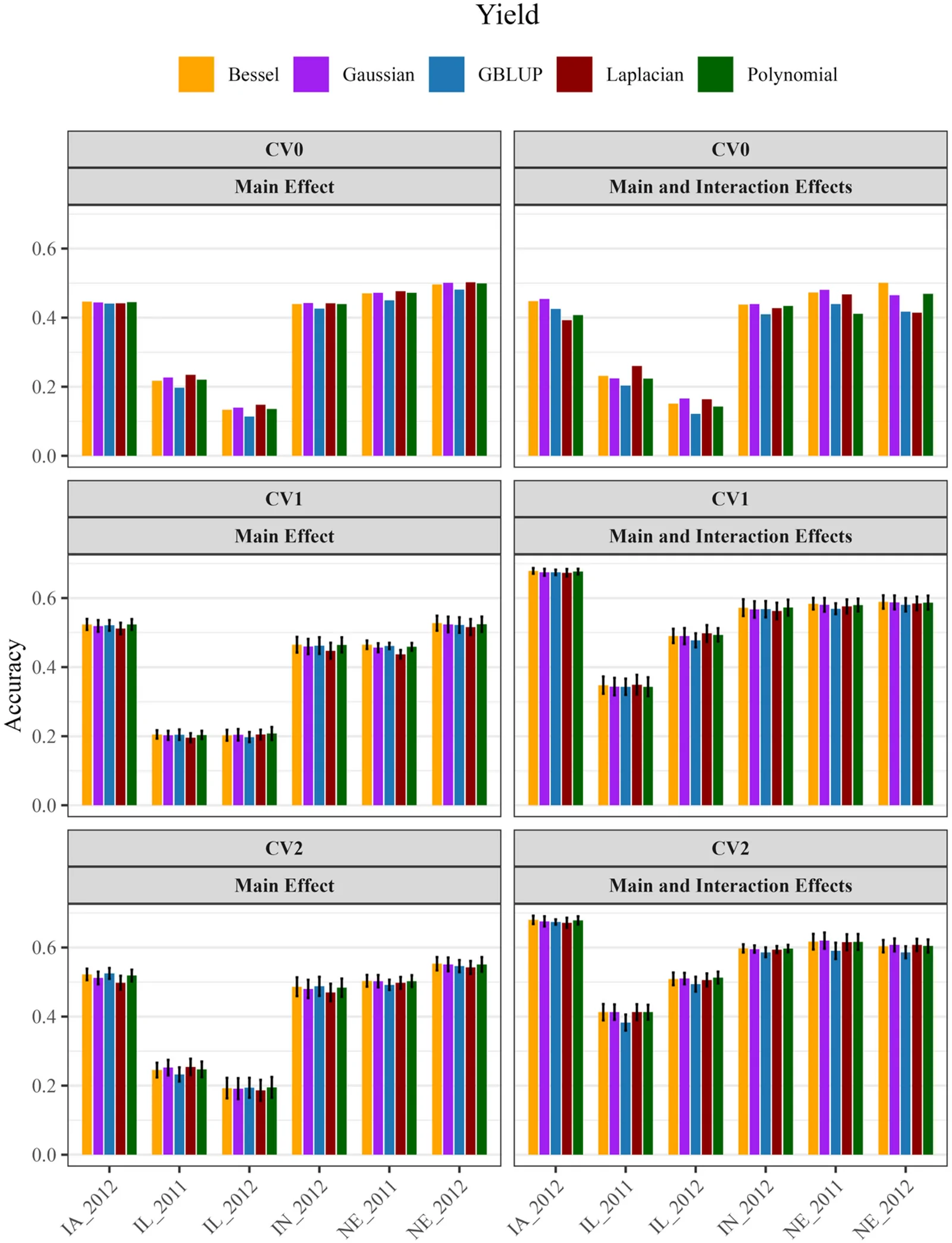
Prediction accuracy for soybean Grain Yield, measured as the correlation between predicted and observed values, evaluated in Iowa (IA, 2012), Illinois (IL, 2011 and 2012), Indiana (IN, 2012), and Nebraska (NE, 2011 and 2012). CV1, cross-validation scheme predicting untested genotypes in observed environments; CV2, cross-validation scheme predicting tested genotypes in observed environments; CV0, cross-validation scheme predicting tested genotypes in unobserved environments. Values represent the mean predictive accuracy across five independent data splits for CV1 and CV2, each evaluated using five-fold cross-validation

Figure 7 shows the prediction accuracy for Yield. Overall, the inclusion of interaction effects resulted in substantial increases in correlation across all CV scenarios. In CV1, when only main effects were considered, correlations varied from 0.20 to 0.55 across models. After including interaction effects, correlations consistently increased, reaching higher values (≈ 0.35 to 0.70). Nonlinear kernels performed similarly to GBLUP across all environments, with no clear advantage of one approach over the others. Similar patterns were observed in CV2. Prediction accuracy varied widely across environments in both scenarios, with the highest values in IA_2012 and the lowest in IL_2011 and IL_2012.

On the other hand, in CV0, overall accuracy was lower compared to CV1 and CV2, with a pronounced reduction in IL_2012. Furthermore, the inclusion of interaction effects resulted in no significant gains, and the differences between models narrowed. Across most environments and in both modeling frameworks, the non-linear kernels generally outperformed GBLUP, with the Bessel and Gaussian kernel showing the most favorable overall performance.

#### Protein content

**Fig. 8:**
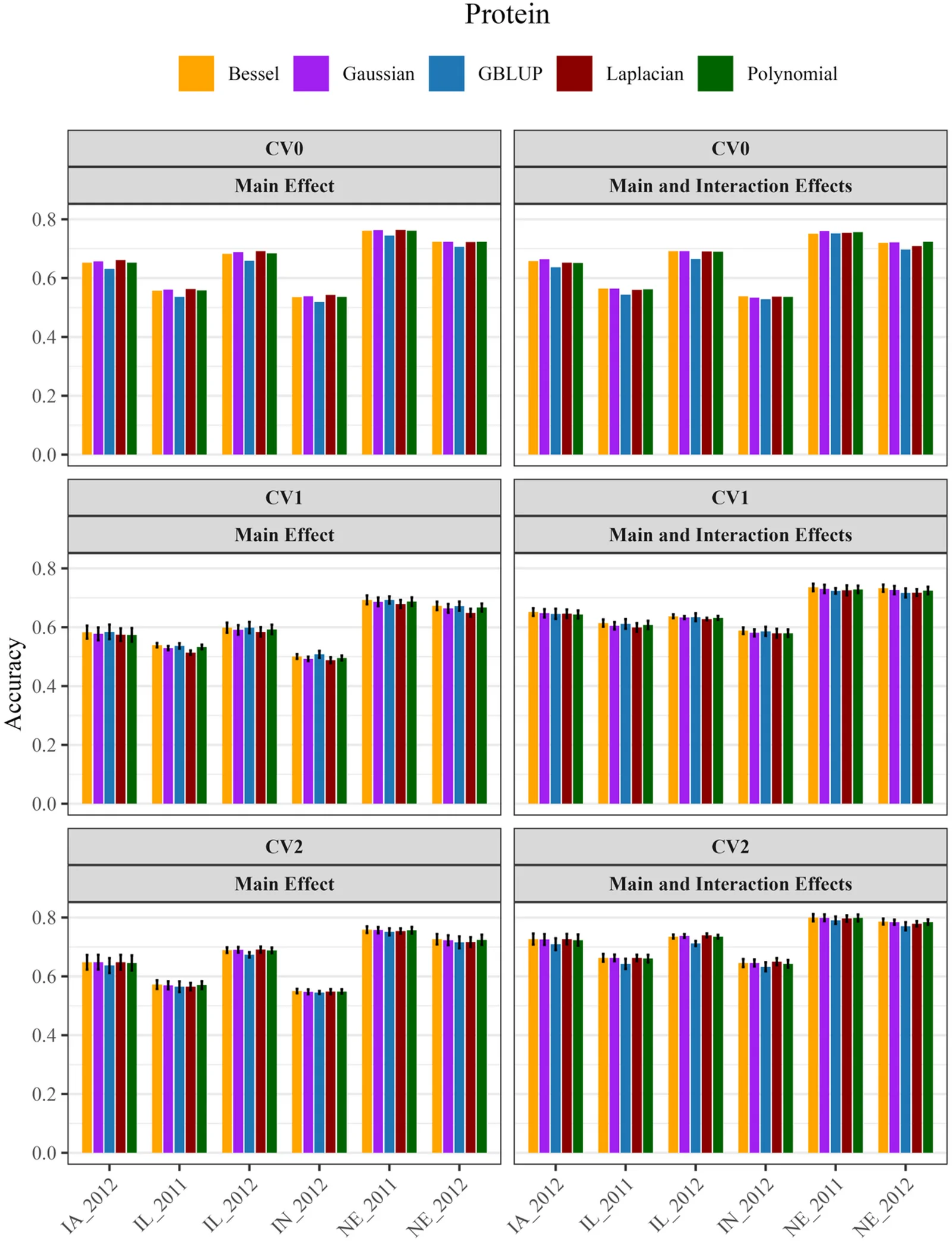
Prediction accuracy for soybean Protein Content, measured as the correlation between predicted and observed values, evaluated in Iowa (IA, 2012), Illinois (IL, 2011 and 2012), Indiana (IN, 2012), and Nebraska (NE, 2011 and 2012). CV1, cross-validation scheme predicting untested genotypes in observed environments; CV2, cross-validation scheme predicting tested genotypes in observed environments; CV0, cross-validation scheme predicting tested genotypes in unobserved environments. Values represent the mean predictive accuracy across five independent data splits for CV1 and CV2, each evaluated using five-fold cross-validation

Prediction accuracy for Protein Content showed consistent improvements across all cross-validation schemes when interaction effects were incorporated (Fig. 8). In CV1, models including only main effects showed moderate accuracy (≈ 0.50 to 0.70 across environments), with better and worse performance in NE_2011 and IN_2012, respectively. The inclusion of interaction effects substantially increased accuracy (≈ 0.59 to 0.72 across environments). Across all environments, kernel methods performed similarly to GBLUP, with no single method consistently outperforming the others.

Under the CV2 scheme, a similar pattern was observed for the main-effects framework (≈ 0.53 to 0.78 across environments). However, when interaction effects were included, the non-linear kernels consistently achieved slightly higher accuracies than GBLUP across most environments (≈ 0.63 to 0.80), although the differences were small.

In addition, under CV0 scheme, there was little difference between the two modeling frameworks, with predictive accuracy generally ranging from approximately 0.54 to 0.78 across environments. In contrast to the other cross-validation schemes, the non-linear kernels showed a more noticeable improvement over GBLUP in both frameworks. However, no consistent differences were observed among the non-linear kernels across environments.

#### Oil content

**Fig. 9:**
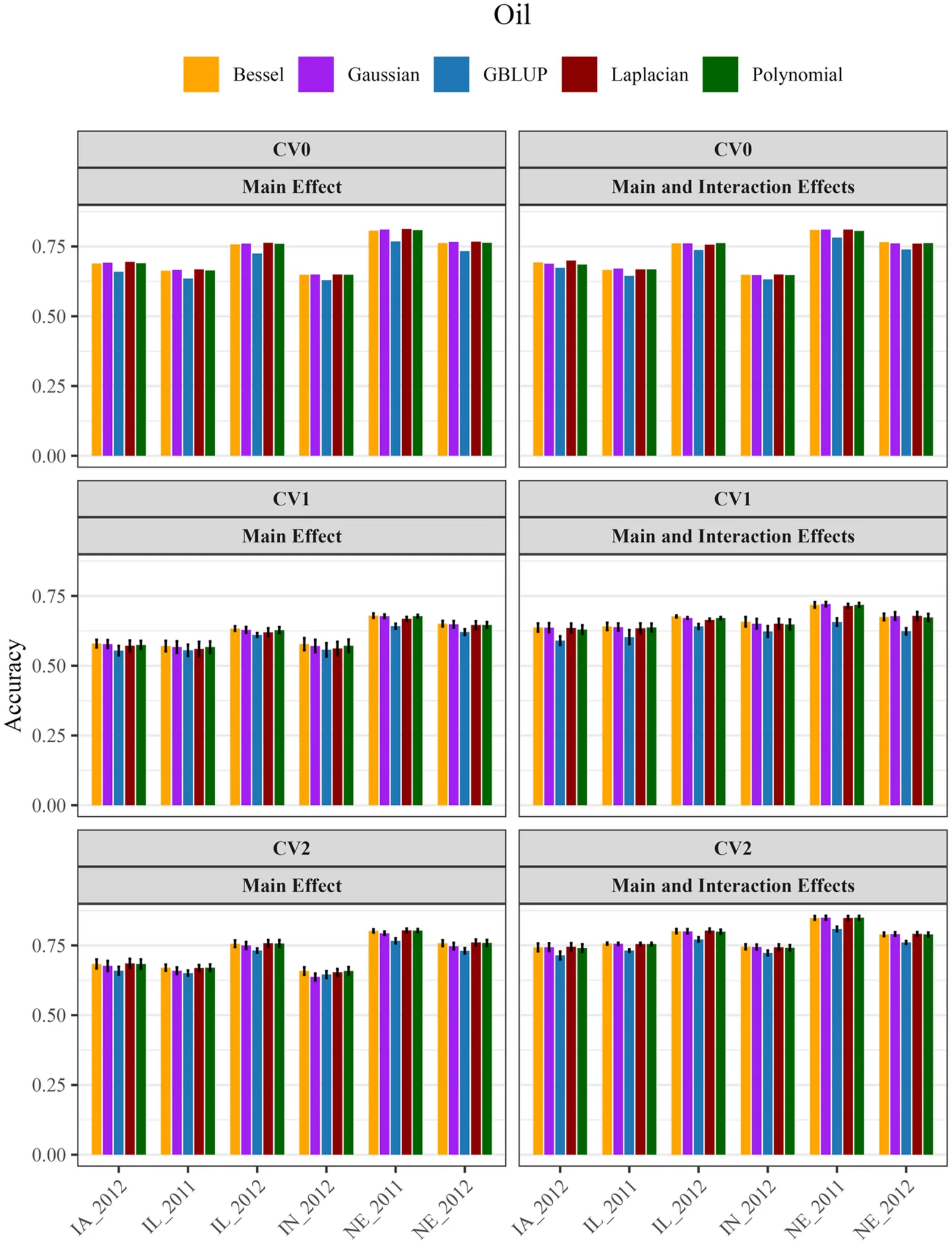
Prediction accuracy for soybean Oil Content, measured as the correlation between predicted and observed values, evaluated in Iowa (IA, 2012), Illinois (IL, 2011 and 2012), Indiana (IN, 2012), and Nebraska (NE, 2011 and 2012). CV1, cross-validation scheme predicting untested genotypes in observed environments; CV2, cross-validation scheme predicting tested genotypes in observed environments; CV0, cross-validation scheme predicting tested genotypes in unobserved environments. Values represent the mean predictive accuracy across five independent data splits for CV1 and CV2, each evaluated using five-fold cross-validation

Prediction accuracy for Oil Content is shown in Fig. 9. In CV1, models with only main effects showed moderate accuracies (≈ 0.53 to 0.73 across environments), with the non-linear kernels outperforming GBLUP. After including interaction effects, the accuracies slightly increased (≈ 0.56 to 0.74 across environments), and the superiority of the non-linear kernels over GBLUP became more evident. Moreover, the differences in accuracy among environments were small compared with Protein and Yield.

In contrast, CV2 showed a clear improvement compared to CV1. Under main effects only, accuracies ranged from ≈ 0.68 to 0.79 across environments, with the best performance in NE_2011 and lower values in IN_2012. In this framework, non-linear kernels modestly outperformed GBLUP. With the inclusion of interaction effects, accuracies increased further (≈ 0.73 to 0.85), and the superiority of the non-linear kernels over GBLUP emerged more clearly, with no significant differences among the non-linear kernels.

Finally, in CV0, the inclusion of interaction effects did not lead to additional gains. Under both modeling strategies, non-linear kernels outperformed GBLUP, with minimal differences among them. Prediction accuracy varied markedly across environments, with NE_2011 showing values above 0.80 and IN_2012 below 0.70.

### Machine learning comparison

The comparison between Random Forest and the best framework (main effects or main and interaction effects) performance of the kernel-based models and GBLUP is presented in Supplementary Fig. 11. Random Forest was competitive only under the CV0 scheme for Protein and Oil, where its predictive accuracies were like those of the non-linear kernels and GBLUP across most environments. In contrast, for Yield, Random Forest consistently showed lower predictive accuracies than the other models under all cross-validation schemes.

### Validation on an independent dataset

In the epistatic traits dataset simulated under different numbers of QTLs, the hyperparameter analysis indicated that the highest predictive accuracies were obtained using specific configurations for each non-linear kernel (Supplementary Fig. 12). For the Laplacian and Gaussian kernel, the best performance was observed with a bandwidth parameter of sigma = 0.001. For the Bessel kernel, the optimal configuration was sigma = 0.1, order = 1, and degree = 2. For the polynomial kernel, the best predictive performance was obtained with degree = 2, scale = 2, and offset = 2. These hyperparameters are chosen for training final model, to compare with GBLUP and Random Forest (Supplementary Fig. 13).

For traits controlled by fewer QTLs, especially 8 QTLs, Random Forest showed the highest predictive accuracy, clearly outperforming the kernel-based models and GBLUP. However, as the number of QTLs increased, the differences among methods became smaller. For 40, 80, and 120 QTLs, the non-linear kernels, particularly Laplacian and Gaussian, showed competitive or slightly superior performance compared with GBLUP and Random Forest. Under highly polygenic architectures, with 240 and 480 QTLs, all methods showed lower and more similar predictive accuracies, although Laplacian, Polynomial, and GBLUP tended to remain among the best-performing approaches.

## Discussion

Nonlinear kernels can implicitly capture complex non-additive genetic patterns through the kernel trick by modifying the genomic covariance structure, rather than by explicitly partitioning epistatic variance components. For GEI modeling, the linear kernel represents interaction patterns using the original genomic covariance structure, whereas non-linear kernels provide a more flexible representation of genomic similarity across environments. Therefore, comparing linear and non-linear GEI kernels allows us to assess whether more complex covariance structures improve the prediction of environment-specific genotype responses.

### Hyperparameter tuning and kernel-specific sensitivity

For model evaluation, kernel hyperparameters were selected based on the mean predictive accuracy across environments and cross-validation schemes using the main-effects framework. Across traits, the results were consistent, with optimal bandwidths of 0.001 for the Laplacian kernel and 0.0001 for the Gaussian kernel. These small bandwidth values indicate that predictive performance was maximized when kernel similarity was concentrated among closely related genotypes, emphasizing local rather than global genetic relationships (De los Campos et al. 2010). Although both kernels emphasize local genetic similarity, they differ in how similarity decays with genetic distance: the Laplacian kernel uses an L1 Norm (Manhattan) (based exponential decay), whereas the Gaussian kernel uses a squared L2 Norm (Euclidean), resulting in a smoother decay of similarity (Schaid 2010) (Supplementary Fig. 1).

The Bessel kernel showed strong sensitivity to the σ parameter, with a clear contrast between σ = 0.1 and the smaller values tested. The best performance observed at σ = 0.01 indicates an optimal balance between global and local similarity, although its advantage over σ = 0.001 was modest. This pattern can be explained by the nature of isotropic kernels, such as the Bessel kernel, which depend exclusively on the distance between individuals (Rasmussen and Williams 2006). Consequently, parameters controlling distance scaling play a central role in defining the covariance structure. In contrast, the order and degree parameters control the functional shape and smoothness of the similarity function, influencing how similarity decays with distance (Gneiting 2002). However, their impact on prediction accuracy was limited, suggesting that once the scale of similarity is appropriately defined by σ, these parameters only provide minor adjustments to the covariance structure.

Otherwise, for the Polynomial kernel, the degree = 2 promotes the best performance over degree = 3, suggesting that a lower-order polynomial was sufficient to capture the underlying structure of the data, whereas higher degrees may have introduced unnecessary complexity. In contrast, the scale and offset parameters induced only subtle variations in performance, suggesting a relatively flat optimization landscape for this specific dataset. This occurs because rescaling and shifting the inner product between individuals do not alter fundamental similarity relationships (Schölkopf and Smola 2002).

Although the Bessel and Polynomial kernels involve more hyperparameters to tune, some of these parameters had little influence on model performance. Specifically, order and degree in the Bessel kernel, as well as offset and scale in the Polynomial kernel, showed limited effects, which may simplify hyperparameter optimization in future studies using similar datasets. In contrast, although the Laplacian and Gaussian kernels were sensitive to sigma, they are easier to optimize because they depend on only one critical hyperparameter, making them more practical candidates for routine applications in breeding programs. Thus, our findings underscore that kernel-specific parameterization is a decisive factor in balancing model accuracy and generalization, as demonstrated by Kismiantini et al. (2022).

### Trait-specific variance components and environmental stability

In this study, all inter-environmental correlations were positive, yet consistent differences emerged across traits. Yield tended to show the lowest correlations across environments, indicating stronger environmental heterogeneity and greater GEI, whereas protein showed intermediate correlations and Oil exhibited the highest and most consistent correlations, suggesting greater stability across environments. This pattern agrees with Yan et al. (2010), who reported that soybean yield was more sensitive to GEI than seed protein and oil.

We found that a high portion of the phenotypic variance for Yield, Protein, and Oil was due to GEI, consistent with Recker et al. (2013). Considering only main effects, little variation was observed among models in the partitioning of variance components. In contrast, clear differences emerged when interaction effects were incorporated. Laplacian, Gaussian, and Bessel kernels showed superior ability to reallocate $\:{\sigma\:}_{\epsilon\:}^{2}$ to $\:{\sigma\:}_{ge}^{2}$ compared to GBLUP, indicating a superior ability of kernels to capture non-linear genetic architectures and heterogeneous GEI patterns. Despite these changes in variance allocation across models, $\:{H}^{2}$ estimates were only marginally affected by the inclusion of interaction effects across all models, being highest for Oil content, followed by Protein content, and lowest for Yield, as reported in the literature (Jaureguy et al. 2011; Recker et al. 2013; Albuquerque et al. 2023). This behavior indicates that the incorporation of GEI primarily reduced the $\:{\sigma\:}_{\epsilon\:}^{2}$ while the $\:{\sigma\:}_{g}^{2}$ was preserved.

The contrasting variance patterns observed among traits, consistent with inter-environmental correlation findings, reinforce that soybean traits differ markedly in their genetic architecture and sensitivity to environmental variation. For Yield, a pronounced redistribution of variance occurs after incorporating GEI, showing stronger environmental dependence and greater influence of GEI, as observed by Bianchi et al. (2020). For Protein, the substantial allocation $\:{\sigma\:}_{ge}^{2}$ indicates that Protein accumulation is also strongly modulated by environmental conditions, although to a lesser extent than Yield. In the case of Oil, the $\:{\sigma\:}_{g}^{2}$ dominance across both modeling strategies reflects the lower sensitivity of this trait to environmental modulation. The relatively smaller $\:{\sigma\:}_{ge}^{2}$ across all models is consistent with results reported for Ethiopian soybean, which show greater environmental influence on Yield performance, followed by Protein, and less on Oil (Gurmu et al. 2010). However, this pattern was not observed in semiarid Brazilian soybean, where the variance of Oil due to $\:{\sigma\:}_{ge}^{2}$ reached nearly 70% (Albuquerque et al. 2023). Therefore, the differences in the sources of phenotypic variation of soybean traits arise from genotype-specific responses interacting with regional climatic conditions, emphasizing the need for tailored GP strategies across production environments.

### Predictive accuracy across traits, models, and cross-validation schemes

The predictive accuracy for Yield, Protein, and Oil soybean was strongly influenced by the effects considered, the model, and the CV scheme used. Overall, Protein and Oil, which exhibited higher environmental correlations, achieved greater predictive accuracy than Yield. These patterns are consistent with findings reported by Ray et al. (2023) and are further supported by Duhnen et al. (2017), who showed that GP explained a greater proportion of phenotypic variation for protein content than for grain yield in soybean, reflecting the higher complexity and stronger environmental influence associated with Yield. Their results also indicate that the inclusion of AAE effects in the GBLUP model led to only slight improvements, explaining 1% to 5% of the proportion of the phenotypic variance.

The inclusion of GEI effects improved predictive accuracy primarily for Yield under the CV1 and CV2 schemes. This result was expected because Yield exhibited lower genetic correlations among environments. Several studies with METs demonstrate that models accounting only for main effects exhibit limited performance under strong environmental heterogeneity, as they do not capture the differential variation of genotypes between environments (Burgueño et al. 2012; Jarquín et al. 2017; Pandey et al. 2020; Crespo-Herrera et al. 2021; Montesinos-López et al. 2022). This improvement can be explained by the covariance structure imposed by the GEI term. In the present formulation, when I_a_ is defined as an identity matrix, the interaction covariance matrix is block-diagonal by environment; therefore, it does not impose direct covariance among interaction effects from different environments. Instead, it models environment-specific genetic deviations by using the genomic covariance among genotypes within each environment. Thus, although the interaction component does not directly borrow information across environments, it allows the model to separate the overall genomic signal from environment-specific deviations, reducing the amount of differential genotypic variation absorbed by the residual term (Lopez-Cruz et al. 2015).

Unlike CV1 and CV2, the explicit inclusion of GEI effects resulted in only marginal improvements under the CV0 scheme. In CV0, predictions are made for environments that are completely absent from the training set. Although the genotypes have been evaluated in other environments, allowing their main genetic effects to be estimated with relatively high precision, the interaction effects for the target environment cannot be directly learned because no phenotypic observations are available. Consequently, predictions rely predominantly on the main genetic effects, limiting the additional contribution of an explicit GEI term.

Regarding the models, for Yield and Protein, non-linear kernels achieved predictive accuracies like those of GBLUP across CV1 and CV2 scenarios. Under CV0, all non-linear kernels slightly outperformed GBLUP under both main effects and main plus interaction effects. In contrast, for Oil, non-linear kernels outperformed GBLUP across all CVs. These differences likely reflect trait-specific genetic architectures, and the ability of each modeling approach captures them. Yield is a highly complex trait, controlled by many loci of small effect and strongly affected by environmental variation and GEI, resulting in lower heritability than seed composition traits. Under these conditions, the infinitesimal assumptions of GBLUP appear sufficient to capture most of the genetic signal, making it a robust and competitive predictor. In contrast, Protein and Oil exhibit higher heritability and more stable genetic control, indicating a stronger genetic signal and simpler marker–trait relationships (Wiggins et al. 2018). Under these conditions, non-linear kernels act as an intermediate approach between linear models such as GBLUP and highly flexible methods like Machine and Deep Learning, being able to capture moderate non-linearities and some interaction patterns without the complexity and data demands of deep architectures, which may not provide additional predictive gains for traits with more stable genetic architecture (Ray et al. 2023). In addition, complex Bayesian models have not consistently outperformed GBLUP for the prediction of Yield and Protein in soybean (early and late lines) (Duhnen et al. 2017), further supporting the effectiveness of parsimonious approaches.

Moreover, for grain yield, although non-linear kernels have the theoretical capacity to account for non-additive genetic effects, their limited improvement over GBLUP suggests that additive effects may be the predominant source of genetic variation in this dataset (Costa et al. 2022). Alternatively, AAE effects may have been less expressive for grain yield than for protein and oil content or were not adequately represented by the selected SNP markers.

Across all CV scenarios, Yield consistently showed reduced accuracy in the IL_2012 environment, while Protein and Oil exhibited recurrent reductions in IL_2011 and IN_2012, highlighting these as consistently more challenging environments for prediction. This pattern reinforces previous findings that extreme or lowly correlated environments with training data drastically limit predictive accuracy, even in sophisticated approaches, underscoring the need for greater environmental representativeness in training datasets to mitigate such limitations (Widener et al. 2021).

### Non-linear kernels as an alternative to machine learning models

Finally, no single non-linear kernel consistently outperformed the others across environments, indicating that the relative performance of the Laplacian, Gaussian, Bessel, and Polynomial kernels was environment dependent. Nevertheless, all non-linear kernels consistently matched or exceeded the predictive performance of GBLUP and also performed comparably to or better than Random Forest across traits and CVs. Although Machine Learning has been widely adopted for GP because of its ability to model complex non-linear relationships without requiring explicit assumptions about the underlying genetic architecture, its advantages are primarily predictive. In contrast, the kernel-based mixed models evaluated in this study not only achieved superior overall predictive performance but also retained the statistical framework of quantitative genetics. This framework enables both non-linear modeling and the direct estimation of the variance components, providing quantitative genetic parameters that support breeding decisions. Therefore, non-linear kernel models represent an attractive alternative for capturing complex genetic relationships while preserving some interpretability and inferential capabilities that are not inherent to most Machine Learning approaches.

### Validation under simulated epistatic genetic architectures

For the epistatic simulation dataset, the best hyperparameter configurations differed from those previously selected for the soybean dataset, indicating that kernel tuning is dataset- and architecture-dependent rather than universally transferable. This pattern was particularly evident for the Bessel kernel: sigma = 0.1 was optimal for the epistatic simulation dataset, but produced the lowest predictive accuracy among the tested values in the soybean dataset. In the simulated data, where the non-linear signal was more controlled and directly associated with QTL interactions, this value likely provided an appropriate scaling of genomic similarities for capturing epistatic patterns. In contrast, the real soybean dataset included GEI, experimental noise, and more diffuse polygenic effects, for which the same sigma may have produced excessive differentiation among genotypes, limiting information sharing across related individuals and reducing predictive accuracy.

Still, the compare between models suggests that the relative advantage of each method depends on the balance between sparsity, non-linearity, and polygenicity in the genetic architecture. When the trait is controlled by a small number of QTLs, the genetic signal is more concentrated in a limited set of loci and interactions. Under this condition, Random Forest can be advantageous because tree-based models are well suited to capturing strong marker effects, threshold-like responses, and interaction structures without requiring an explicit parametric specification (González-Camacho et al. 2018; Costa et al. 2022; Celeri et al. 2026).

As the number of QTLs increased, the predictive signal became more distributed across the genome. In this context, the advantage of Random Forest decreased because individual marker effects became weaker and more diffuse, making it harder for tree-based splits to consistently identify the most informative predictors. Kernel-based models, especially Laplacian and Gaussian kernels, became more competitive because they model genetic similarity among individuals rather than selecting specific markers (Morota and Gianola 2014; Montesinos-López et al. 2021).

Under highly polygenic architectures, the convergence among methods suggests that much of the predictable genetic signal can be captured by broad genomic similarity. In this situation, the marginal benefit of more flexible non-linear models becomes smaller, and GBLUP can remain competitive because additive genomic relationships approximate a large portion of the signal generated by many small-effect loci (VanRaden 2008).

In addition, the competitive performance of the Laplacian kernel across all genetic architectures, together with the favorable performance of the Gaussian kernel for traits controlled by 40, 80, and 120 QTLs, highlights the potential of non-linear kernels to capture epistatic effects distributed across multiple genomic regions. Non-linear RKHS kernels transform marker-derived similarities into higher-dimensional representations, thereby accommodating complex marker interactions without requiring the explicit estimation of every pairwise or higher-order epistatic effect. In particular, Jiang and Reif (2015) demonstrated that the Gaussian kernel implicitly incorporates AAE relationships of multiple orders, providing a theoretical basis for its ability to model epistasis in GP. Similarly, Akdemir and Jannink (2015) emphasized that Gaussian and other non-linear kernels can incorporate both additive and complex epistatic marker effects, and reported that such models may outperform linear kernels when non-additive genetic variation contributes meaningfully to the trait. While the Gaussian kernel has become one of the most widely adopted for GP, our results indicate that the Laplacian kernel represents a promising alternative in future GP studies.

### Non-linear kernels for modeling complex genetic architectures and GEI

While the Polynomial (Montesinos-López et al. 2021, 2022) and Gaussian (Cuevas et al. 2016, 2017; Sousa et al. 2017; Montesinos-López et al. 2022) kernels have already demonstrated the ability to match or surpass GBLUP, the Laplacian and Bessel kernels remain underexplored in the GP literature. Whilst Polynomial and Bessel kernels of varying orders can model progressively complex marker interactions, the Laplacian and Gaussian kernels can excel at representing intricate epistatic relationships (Akdemir and Jannink 2015). Thus, this study demonstrates that incorporating non-linear kernel functions can improve the modeling of complex genetic architectures and GEI, leading to more robust GP in multi-environment scenarios.

## Conclusion

In this study, we compared GBLUP, Random Forest, and four non-linear kernels (Laplacian, Gaussian, Bessel, and Polynomial) for multi-environment genomic prediction across Yield, Protein, and Oil. Overall, predictive performance was trait-dependent, reflecting differences in genetic architecture and the magnitude of GEI. Grain Yield remained the most challenging trait due to its greater environmental sensitivity, whereas Oil and Protein showed more stable predictions across environments.

Across all traits, the non-linear kernels consistently matched or outperformed GBLUP, with the greatest advantage observed for Oil and under the CV0 scheme. No single non-linear kernel emerged as the best performer across all environments, indicating that kernel choice should be guided by the characteristics of the target dataset rather than by a universally optimal kernel. The explicit modeling of GEI substantially improved predictive accuracy for Yield under CV1 and CV2 but provided only limited gains under CV0, where predictions relied primarily on genetic relationships learned from the observed environments.

In the epistatic trait simulated dataset, the Laplacian kernel consistently achieved high predictive accuracy across all scenarios, while the Gaussian kernel performed particularly well under the oligogenic traits, highlighting the potential of non-linear kernels for genomic prediction. These findings identify the Laplacian kernel as a robust and promising alternative to the widely used Gaussian kernel.

Beyond their competitive predictive performance, the non-linear kernel models retained the mixed-model framework, allowing the estimation of variance components and other quantitative genetic parameters. This represents an important advantage over conventional Machine Learning approaches, which are primarily predictive and do not directly support quantitative genetic inference. Therefore, non-linear kernel models provide a practical compromise between linear mixed models and Machine Learning approaches, combining the flexibility to model complex genetic relationships with the interpretability required for plant breeding applications.

## Supplementary Information

Below is the link to the electronic supplementary material.

Supplementary Material 1

## Data Availability

A repository containing all the scripts and documentation to reproduce the analysis is available at: https://wwanessa13.github.io/nlkernel-soybean/.
