# Supplementary material for: Non-linear kernel methods for genomic prediction of soybean yield and quality in multi-environment trials
Source: Theor Appl Genet. 2026 Sep 23;139(10):277. doi: 10.1007/s00122-026-05387-3 (PMC13601130; doi:10.1007/s00122-026-05387-3)
Supplement: Supplementary file 1 [file 122_2026_5387_MOESM1_ESM.docx]

**Supplementary Information**

**Supplementary Figure 1:** Representative shapes of (A) Laplacian kernel and (B) Gaussian kernel functions, where *x* represents the distance between x_i_ and x_j_. The curves illustrate different values of the bandwidth parameter σ (0.1, 0.01, 0.001, and 0.0001), highlighting how σ controls the rate of decay, smoothness, and degree of locality of the similarity function.

**Supplementary Figure 2:** Representative shapes of the Bessel kernel function, where *x* represents the distance between x_i_ and x_j_. The panel illustrates functional forms resulting from different combinations of hyperparameters tested (degree, order, and sigma). The J₀ and J₁ were approximated using the Taylor series up to a finite order. The curves demonstrate how these parameters combinations affect symmetry, curvature, and local behavior of the kernel, highlighting the flexibility of the Bessel kernel in capturing complex, non-linear similarity patterns.

**Supplementary Figure 3:** Representative shapes of the Polynomial kernel functions, where x represents the distance between x_i_ and x_j_. The panel illustrates functional forms resulting from different combinations of degree, scale, and offset parameters. The curves show how variations in these parameters affect curvature, growth rate, and symmetry of the kernel function, highlighting the flexibility of the Polynomial kernel in modeling diverse non-linear similarity structures.

**Supplementary Figure 4.** Phenotypic analysis for yield in Iowa (IA_2012), Illinois (IL_2011, IL_2012), Indiana (IN_2012), and Nebraska (NE_2011, NE_2012). (A) Histograms of grain yield distributions across environments. (B) Pairwise phenotypic correlation matrix between the grain yield of genotypes in different environments, highlighting Genotype-by-Environment Interaction (GEI) patterns.

**Supplementary Figure 5.** Phenotypic analysis for protein in Iowa (IA_2012), Illinois (IL_2011, IL_2012), Indiana (IN_2012), and Nebraska (NE_2011, NE_2012). (A) Histograms of protein content distributions across environments. (B) Correlation matrix between the protein content of genotypes in different environments, highlighting Genotype-by-Environment Interaction (GEI) patterns.

**Supplementary Figure 6.** Phenotypic analysis for oil in Iowa (IA_2012), Illinois (IL_2011, IL_2012), Indiana (IN_2012), and Nebraska (NE_2011, NE_2012). (A) Histograms of oil content distributions across environments. (B) Correlation matrix between the oil content of genotypes in different environments, highlighting Genotype-by-Environment Interaction (GEI) patterns.

**Supplementary Figure 7.** Predictive accuracy of the Laplacian kernel under different bandwidth (σ) values across environments and cross-validation schemes (CV0, CV1, and CV2) for Yield, Protein, and Oil, under the main effects framework.

**Supplementary Figure 8.** Predictive accuracy of the Gaussian kernel under different bandwidth (σ) values across environments and cross-validation schemes (CV0, CV1, and CV2) for Yield, Protein, and Oil, under the main effects framework.

**Supplementary Figure 9.** Predictive accuracy of the Bessel kernel under different sigma, order, and degree values across environments and cross-validation schemes (CV0, CV1, and CV2) for Yield, Protein, and Oil, under the main effects framework.

**Supplementary Figure 10.** Predictive accuracy of the Polynomial kernel under different degree, offset, and scale values across environments and cross-validation schemes (CV0, CV1, and CV2) for Yield, Protein, and Oil, under the main effects framework.

**Supplementary Table 1:** Estimates of variance components varied among traits and models. G = $\sigma_{\boldsymbol{g}}^{\boldsymbol{2}}$; GE = $\sigma_{\boldsymbol{ge}}^{\boldsymbol{2}}$; e = $\sigma_{\varepsilon}^{\boldsymbol{2}}$.

| **Trait** | **Model** | **Effect** | **Component** | **Value** |
| --- | --- | --- | --- | --- |
| Yield | GBLUP | Main | G | 74646.09 |
| Yield | GBLUP | Main | e | 335465.71 |
| Protein | GBLUP | Main | G | 0.58 |
| Protein | GBLUP | Main | e | 0.77 |
| Oil | GBLUP | Main | G | 0.23 |
| Oil | GBLUP | Main | e | 0.22 |
| Yield | Laplacian | Main | G | 72970.81 |
| Yield | Laplacian | Main | e | 323758.25 |
| Protein | Laplacian | Main | G | 0.60 |
| Protein | Laplacian | Main | e | 0.72 |
| Oil | Laplacian | Main | G | 0.25 |
| Oil | Laplacian | Main | e | 0.20 |
| Yield | Gaussian | Main | G | 57894.87 |
| Yield | Gaussian | Main | e | 329717.95 |
| Protein | Gaussian | Main | G | 0.58 |
| Protein | Gaussian | Main | e | 0.72 |
| Oil | Gaussian | Main | G | 0.25 |
| Oil | Gaussian | Main | e | 0.20 |
| Yield | Bessel | Main | G | 76409.92 |
| Yield | Bessel | Main | e | 327088.57 |
| Protein | Bessel | Main | G | 0.60 |
| Protein | Bessel | Main | e | 0.73 |
| Oil | Bessel | Main | G | 0.25 |
| Oil | Bessel | Main | e | 0.20 |
| Yield | Polynomial | Main | G | 77362.80 |
| Yield | Polynomial | Main | e | 325636.07 |
| Protein | Polynomial | Main | G | 0.60 |
| Protein | Polynomial | Main | e | 0.73 |
| Oil | Polynomial | Main | G | 0.25 |
| Oil | Polynomial | Main | e | 0.20 |
| Yield | GBLUP | Main and Interaction | G | 64280.44 |
| Yield | GBLUP | Main and Interaction | e | 237635.74 |
| Yield | GBLUP | Main and Interaction | GE | 249028.20 |
| Protein | GBLUP | Main and Interaction | G | 0.58 |
| Protein | GBLUP | Main and Interaction | e | 0.58 |
| Protein | GBLUP | Main and Interaction | GE | 0.28 |
| Oil | GBLUP | Main and Interaction | G | 0.25 |
| Oil | GBLUP | Main and Interaction | e | 0.16 |
| Oil | GBLUP | Main and Interaction | GE | 0.05 |
| Yield | Laplacian | Main and Interaction | G | 43661.37 |
| Yield | Laplacian | Main and Interaction | e | 86840.36 |
| Yield | Laplacian | Main and Interaction | GE | 545038.19 |
| Protein | Laplacian | Main and Interaction | G | 0.51 |
| Protein | Laplacian | Main and Interaction | e | 0.24 |
| Protein | Laplacian | Main and Interaction | GE | 1.15 |
| Oil | Laplacian | Main and Interaction | G | 0.23 |
| Oil | Laplacian | Main and Interaction | e | 0.06 |
| Oil | Laplacian | Main and Interaction | GE | 0.31 |
| Yield | Gaussian | Main and Interaction | G | 54895.86 |
| Yield | Gaussian | Main and Interaction | e | 68975.30 |
| Yield | Gaussian | Main and Interaction | GE | 187268.64 |
| Protein | Gaussian | Main and Interaction | G | 0.57 |
| Protein | Gaussian | Main and Interaction | e | 0.16 |
| Protein | Gaussian | Main and Interaction | GE | 0.38 |
| Oil | Gaussian | Main and Interaction | G | 0.24 |
| Oil | Gaussian | Main and Interaction | e | 0.04 |
| Oil | Gaussian | Main and Interaction | GE | 0.10 |
| Yield | Bessel | Main and Interaction | G | 62469.55 |
| Yield | Bessel | Main and Interaction | e | 197237.76 |
| Yield | Bessel | Main and Interaction | GE | 357199.85 |
| Protein | Bessel | Main and Interaction | G | 0.59 |
| Protein | Bessel | Main and Interaction | e | 0.47 |
| Protein | Bessel | Main and Interaction | GE | 0.47 |
| Oil | Bessel | Main and Interaction | G | 0.25 |
| Oil | Bessel | Main and Interaction | e | 0.12 |
| Oil | Bessel | Main and Interaction | GE | 0.20 |
| Yield | Polynomial | Main and Interaction | G | 59033.62 |
| Yield | Polynomial | Main and Interaction | e | 186848.05 |
| Yield | Polynomial | Main and Interaction | GE | 127232.31 |
| Protein | Polynomial | Main and Interaction | G | 0.60 |
| Protein | Polynomial | Main and Interaction | e | 0.46 |
| Protein | Polynomial | Main and Interaction | GE | 0.19 |
| Oil | Polynomial | Main and Interaction | G | 0.25 |
| Oil | Polynomial | Main and Interaction | e | 0.12 |
| Oil | Polynomial | Main and Interaction | GE | 0.05 |

**Supplementary Figure 11.** Predictive accuracy of Random Forest and the best-performing versions of the kernel-based models (Laplacian, Gaussian, Bessel, and Polynomial) and GBLUP across environments for Yield, Protein, and Oil under the CV0, CV1, and CV2 cross-validation schemes. For each kernel-based model and GBLUP, the framework (main effects or main and interaction effects) with the highest predictive accuracy was selected for comparison with Random Forest. Values represent the mean predictive accuracy across five independent data splits for CV1 and CV2, each evaluated using five-fold cross-validation.

**Supplementary Figure 12.** Predictive accuracy of Laplacian, Gaussian, Bessel, and Polynomial kernels across hyperparameter combinations and epistatic traits controlled by different numbers of QTLs.

**Supplementary Figure 13.** Predictive accuracy of non-linear kernel methods (Laplacian, Gaussian, Bessel, and Polynomial), GBLUP, and Random Forest for genetically controlled epistatic traits simulated with different numbers of QTLs. Values represent the mean predictive accuracy across five independent data splits, each evaluated using five-fold cross-validation.
